# Associations of brominated flame retardants exposure with chronic obstructive pulmonary disease: A US population-based cross-sectional analysis

**DOI:** 10.3389/fpubh.2023.1138811

**Published:** 2023-03-10

**Authors:** Lu Han, Qi Wang

**Affiliations:** Department of Obstetrics and Gynecology, First Affiliated Hospital, Xi'an Jiaotong University, Xi'an, China

**Keywords:** brominated flame retardants, chronic obstructive pulmonary disease, weighted quantile sum model, quantile-based g-calculation model, NHANES

## Abstract

**Backgrounds:**

Whether there existed an association between brominated flame retardants (BFRs) and chronic obstructive pulmonary disease (COPD) prevalence in humans is still a mystery.

**Objective:**

To investigate the association between serum single or mixture BFRs and COPD prevalence.

**Methods:**

Data of 7,591 participants from NHANES 2007–2016 was utilized. Serum BFRs, including PBDE-28, PBDE-47, PBDE-85, PBDE-99, PBDE-100, PBDE-154, PBDE-183, PBDE-209, and PBB-153 were enrolled. The survey-weighted generalized logistic regression model, restricted cubic splines (RCS), weighted quantile sum (WQS) regression, and quantile-based g-computation (QGC) analysis were performed.

**Results:**

After adjustment for all confounding factors, log-transformed continuous serum PBDE-28 (OR: 1.43; 95% CI: 1.10–1.85; *P* = 0.01), PBDE-47 (OR: 1.39; 95% CI: 1.11–1.75; *P* = 0.005), PBDE-85 (OR: 1.31; 95% CI: 1.09–1.57; *P* = 0.005), PBDE-99 (OR: 1.27; 95% CI: 1.05–1.54; *P* = 0.02), PBDE-100 (OR: 1.33; 95% CI: 1.08–1.66; *P* = 0.01), PBDE-154 (OR: 1.29; 95% CI: 1.07–1.55; *P* = 0.01), PBDE-183 (OR: 1.31; 95% CI: 1.04–1.66; P = 0.02), and PBB-153 (OR: 1.25; 95% CI: 1.03–1.53; *P* = 0.03) were positively correlated with the prevalence of COPD. Restricted cubic splines curves displayed that PBDE-209 was significantly associated with CPOD in an inverted U-shape (*P* = 0.03). A significant interaction between being male and a high prevalence of COPD was observed for PBDE-28 (*P* for interaction <0.05), PBDE-47 (P for interaction <0.05), PBDE-85 (*P* for interaction <0.05), PBDE-99 (*P* for interaction <0.05), PBDE-100 (*P* for interaction <0.05), and PBB-153 (*P* for interaction < 0.05). Mixture BFRs exposure was positively associated with COPD prevalence in WQS regression (OR: 1.40; 95% CI: 1.14–1.72, *P* = 0.002) and in QGC analysis (OR: 1.49; 95% CI: 1.27–1.74, *P* < 0.001).

**Conclusions:**

Our study confirms that individual and mixture BFRs had positive associations with COPD, and further studies are required in larger-scale populations.

## Introduction

Chronic obstructive pulmonary disease (COPD) is a common, preventable, and treatable chronic airway disease characterized by persistent respiratory symptoms and airflow limitation. The onset of COPD is usually associated with high exposure to harmful particles or gases. Chronic obstructive pulmonary disease severely impacts patients' health and quality of life and is a significant cause of death. From 1990 to 2017, the prevalence of COPD increased by 5.9% worldwide, and an estimated 298 million patients with COPD accounted for an average of 41.9 deaths per 100,000 people in the world in 2017 (1). The World Health Organization reported that COPD is the third leading cause of death worldwide, and the annual death toll will reach 4.4 million and 90% of COPD deaths by 2040 (2, 3).

Brominated flame retardants (BFRs), including polybrominated diphenyl ethers (PBDEs) and polybrominated biphenyls (PBBs), are widely used in a range of consumer goods such as furniture, carpets, building materials, toys, and electronics to avoid fire risks (4). For not adhering permanently to products, BFRs could be released into the surrounding environment, like air, dust, food, and water (5). Due to their persistence and bioaccumulation characteristics, humans are still exposed to BFRs for the enormous storage and increased recycling of large quantities of BFRs-containing products, despite a ban on their use for many years (6, 7). Human exposure to PBDEs and PBBs is likely to continue for many years, although they cease to be produced and used.

Numerous researchers have confirmed that BFRs are present in the respiratory tract of animals and humans and affect bronchial epithelial cells, including inhibiting cell viability, activating cell apoptosis, inducing DNA damage, and promoting inflammatory and oxidative stress status (8–13). Besides, the changes mentioned above in the respiratory tract also have a role in the development of COPD (14, 15). In addition, not only individual BFR was confirmed to influence human health, but also exposure to BFRs mixture was positively associated with diseases including metabolic syndrome, hypertriglyceridemia, and abdominal obesity (16).

However, there were still no studies investigating the association between single or mixture BFRs with COPD prevalence in humans. Therefore, we extracted data from the National Health and Nutrition Examination Survey (NHANES) to explore the associations of individual and mixture BFRs exposure with COPD prevalence in US adults using different models, including survey-weighted generalized logistic regression model, restricted cubic splines (RCS), weighted quantile sum (WQS) regression, and the quantile-based g-computation (QGC) analysis.

## Methods

### Study population

The NHANES is a cross-sectional, nationally representative study that measures the health and nutritional status of non-institutionalized U.S. populations. Our analysis investigated data from the five consecutive NHANES cycles during 2007–2016. Participants younger than 18, those missing data on critical confounders [such as education, family income to poverty (FIR), body mass index (BMI), smoking, and diabetes], and those without BRFs or COPD data were excluded from the study.

### Exposure assessment

Detailed instructions on collecting, storing, and processing blood specimens were provided in the NHANES Laboratory Procedures Manual. Brominated flame retardants concentrations were determined using an automated liquid/liquid extraction and isotope dilution—ultra-performance liquid chromatography-tandem mass spectrometry method. The NHANES dataset contained BRFs including PBDE-17, PBDE-28, PBDE-47, PBDE-66, PBDE-85, PBDE-99, PBDE-100, PBDE-153, PBDE-154, PBDE-183, PBDE-209, and PBB-153. PBDE-17, PBDE-66, and PBDE-153 were excluded from the analysis because more than 50% of values were below the lower limit of detection (LLOD). Concentrations less than the LLOD were replaced with the LLOD divided by the square root of 2.

### Definition of COPD

Chronic obstructive pulmonary disease was defined by fulfilling any of the following criteria: (1) post-bronchodilator forced expiratory volume in 1 second (FEV1)/forced vital capacity (FVC) ratio <0.7; (2) self-reported COPD, emphysema, or chronic bronchitis; and (3) older than 40 years old, with a history of smoking or bronchitis, and taking any of the following medications: mast cell stabilizers, inhaled corticosteroids, leukotriene modifying agents, or selective phosphodiesterase four inhibitors.

### Covariates

The potential confounding variables included age (continuous variable), gender (male and female), race/ethnicity (Non-Hispanic Black, Non-Hispanic White, Mexican American, and Other), FIR (low income, and middle or high income), BMI (underweight or normal weight, overweight, and obesity), smoking status (former, now, never), physical activity (none, and moderate or vigorous), hypertension (yes and no), and diabetes (yes, borderline, and no). Participants with FIR ≤ 1.3 were categorized as low-income, whereas those with a FIR of 1.3 or higher were classified as middle (1.3–3.5) or high-income (≥3.5). Body mass index was categorized into underweight or normal weight (25 kg/m^2^), overweight (25–30 kg/m^2^), and obese (30 kg/m^2^) groups. Based on standard smoking questionnaires, the smoking status was categorized into never smokers (not smoked as many as 100 cigarettes in their lifetime), former smokers (smoked as many as 100 cigarettes but did not smoke cigarettes currently), and current smokers (currently smoked cigarettes every day or some days). To determine whether a participant had hypertension, we used the following criteria: (1) self-reported hypertension; (2) taking anti-hypertension medications; (3) an average systolic pressure of at least 140 mm Hg or an average diastolic pressure of at least 90 mm Hg. The following criteria were utilized to determine whether a participant had diabetes: (1) self-reported diabetes; (2) use of anti-diabetes medications; (3) glycohemoglobin HbA1c higher than 6.5%; (4) fasting glucose of at least 7.0 mmol/l; (5) random blood glucose of at least 11.1 mmol/l; (6) OGTT blood glucose of at least 11.1 mmol/l. Besides, participants with impaired fasting glycemia and impaired glucose tolerance were diagnosed with borderline diabetes.

### Statistical analyses

In the descriptive analysis, continuous variables were expressed by the mean and standard deviation (SD), while categorical variables were presented as frequency and percentage. The Chi-square test was used for categorical data, and the *T*-test was utilized for continuous variables to compare group differences between the COPD group and the non-COPD group. For skewed distributions, we used log-transformed serum BRFs concentrations in this study and then categorized them into four quartiles.

In order to assess potential associations between individual BFR and COPD, we used the survey-weighted generalized logistic regression models adjusting for confounding variables to calculate odds ratios (ORs) and corresponding 95% confidence intervals (CIs). Given the complex, multistage sampling design in the NHANES, “sum of adjusted subsample B weights” or “adjust sum sampling weights in same pool” were used in this study according to National Center for Health Statistics guidance. Model 1 was controlled for age and gender. Model 2 was further adjusted for race/ethnicity, FIR, and BMI based on Model 1. Model 3 was further adjusted for physical activity, smoking status, hypertension, and diabetes based on Model 2. In the statistical mode, log-transformed serum BRFs were created as a continuous and categorical variable with the lowest quartile as the reference. The potential non-linear associations between individual BFR and COPD were explored by RCSs with three knots. We conducted analyses stratified by sex (male/female) and age (<60 or ≥60 years) and then explored the interaction analyses. Since smoking is an important confounding factor, sensitivity analysis was performed using cotinine levels as the covariate rather than the categories of smoking status to validate the robustness of results.

After adjusting for all confounders, we used the WQS regression and the QGC analysis to examine the overall effect of BRFs exposure on COPD. The WQS regression model has been commonly used to explore the directional, linear, and additive effects of mixed chemicals exposures on human health outcomes (17). The training set (40%) and validation set (60%) were randomly divided among this model. The overall effect of BFRs was investigated by calculating regression coefficients using 1,000 bootstrapped samples from the training dataset. QGC analysis, combining the inferential simplicity of WQS regression with the flexibility of g-computation, was also conducted to assess the cumulative effects of BRFs on COPD (18). In this study, 1,000 bootstrapping iterations were performed on the mixture slope and overall model confidence.

All analyses were conducted in this study using R software version 4.1.2. (Core Team, Vienna, Austria), and a two-sided *P*-value of < 0.05 was considered statistically significant.

## Results

### Basic characteristics

Ultimately, a total of 7,591 participants were included from the NHANES 2007–2016, and 395 (5%) participants had a diagnosis of COPD (Figure S1). Characteristics of the participants enrolled in this study are shown in Table 1. In addition, age, race, recreational activities, smoking status, diabetes, hypertension, PBDE-28, PBDE-47, PBDE-85, PBDE-99, PBDE-100, PBDE-154, and PBB-153 were significant differences between participants with COPD and those without COPD.

**Table 1:** Weighted characteristics of the study participants.

	**Overall**	**Non-CPOD**	**COPD**	**P-value**
	**(*****n*** = **7,591)**	**(*****n*** = **7,268)**	**(*****n*** = **395)**	
**Age [years, mean (SD)]**	47.31 (0.36)	46.63 (0.37)	59.82 (0.79)	<0.0001
**Gender (%)**				0.18
Female	3,886 (51.19)	3,719 (51.72)	167 (46.25)	
Male	3,705 (48.81)	3,477 (48.28)	228 (53.75)	
**Race/Ethnicity (%)**				<0.0001
Mexican American	1,121 (14.77)	1,104 (8.28)	17 (1.36)	
Non-Hispanic Black	1,597 (21.04)	1,530 (11.55)	67 (7.00)	
Non-Hispanic White	3,238 (42.66)	2,981 (67.33)	257 (83.61)	
Other Race	1,635 (21.54)	1,581 (12.84)	54 (8.03)	
**FIR (%)**				0.34
≤ 1.3	2,486 (32.75)	2,340 (21.94)	146 (26.10)	
1.3–3.5	2,297 (30.26)	2,191 (42.48)	106 (38.42)	
≥3.5	2,808 (36.99)	2,665 (35.58)	143 (35.48)	
**Vigorous/Moderate recreational activities (%)**				<0.0001
Yes	3,659 (48.2)	3,525 (55.81)	134 (40.85)	
No	3,932 (51.8)	3,671 (44.19)	261 (59.15)	
**Smoking status (%)**				<0.0001
Now	1,547 (20.38)	1,398 (19.37)	149 (37.89)	
Former	1,853 (24.41)	1,672 (23.32)	181 (44.89)	
Never	4,191 (55.21)	4,126 (57.31)	65 (17.22)	
**Body mass index (%)**				0.37
Underweight or Normal	2,212 (29.14)	2,106 (31.11)	106 (26.06)	
Overweight	2,865 (37.74)	2,710 (35.96)	155 (40.49)	
Obesity	2,514 (33.12)	2,380 (32.93)	134 (33.46)	
**Hypertension (%)**				<0.0001
Yes	4,375 (57.63)	4,221 (63.59)	154 (46.73)	
No	4,375 (57.63)	4,221 (63.59)	154 (46.73)	
**Diabetes (%)**				<0.001
Yes	1,465 (19.3)	1,338 (13.50)	127 (26.03)	
Borderline	667 (8.79)	621 (8.62)	46 (11.96)	
No	5,459 (71.91)	5,237 (77.87)	222 (62.01)	
**Exposures [pg/g, mean (SD)]**				
PBDE28	7.83 (0.13)	7.72 (0.12)	9.88 (0.51)	<0.0001
PBDE47	145.13 (2.75)	143.15 (2.55)	181.82 (11.00)	<0.001
PBDE85	3.21 (0.07)	3.16 (0.07)	3.96 (0.27)	0.002
PBDE99	31.12 (0.74)	30.70 (0.69)	38.77 (2.86)	0.003
PBDE100	31.00 (0.56)	30.60 (0.52)	38.44 (2.42)	<0.001
PBDE154	2.88 (0.06)	2.84 (0.06)	3.56 (0.24)	0.002
PBDE183	1.79 (0.06)	1.78 (0.05)	1.96 (0.14)	0.19
PBDE209	18.34 (0.47)	18.32 (0.45)	18.77 (1.81)	0.79
PBB153	27.32 (0.90)	26.60 (0.90)	40.63 (2.71)	<0.0001

### Associations of individual BFRs exposure with COPD prevalence

**Figure 1:**
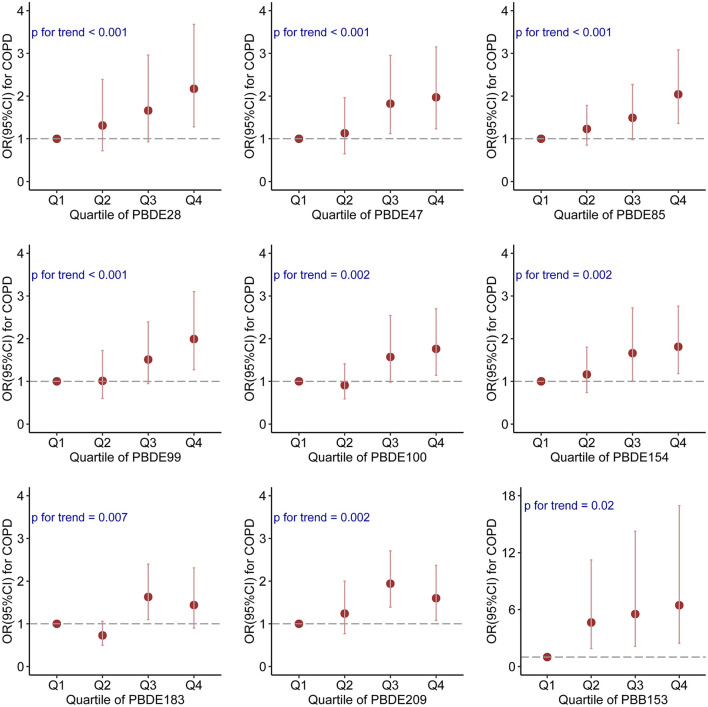
The associations between quartiles of individual BFR and COPD prevalence.

The survey-weighted generalized logistic regression models were used to analyze the associations between individual BFRs exposure and COPD prevalence. After adjustment for all confounding factors, log-transformed continuous serum PBDE-28 (OR: 1.43; 95% CI: 1.10–1.85; *P* = 0.01), PBDE-47 (OR: 1.39; 95% CI: 1.11–1.75; *P* = 0.005), PBDE-85 (OR: 1.31; 95% CI: 1.09–1.57; *P* = 0.005), PBDE-99 (OR: 1.27; 95% CI: 1.05–1.54; *P* = 0.02), PBDE-100 (OR: 1.33; 95% CI: 1.08–1.66; *P* = 0.01), PBDE-154 (OR: 1.29; 95% CI: 1.07–1.55; *P* = 0.01), PBDE-183 (OR: 1.31; 95% CI: 1.04–1.66; *P* = 0.02), and PBB-153 (OR: 1.25; 95% CI: 1.03–1.53; *P* = 0.03) were positively correlated with the prevalence of COPD (Table 2). When divided into quantiles, higher quantiles of PBDE-28, PBDE-47, PBDE-85, PBDE-99, PBDE-100, PBDE-154, PBDE-183, PBDE-209, and PBB-153 were associated with increased odds of COPD (all *P*_fortrend_ < 0.001) (Figure 1). After using cotinine levels as the covariate rather than the categories of smoking status, the associations between BRFs and COPD prevalence were not altered (Table S1).

**Table 2:** Association between log-transformed serum BFRs and prevalence of COPD.

	**Model 1**	**P-value**	**Model 2**	**P-value**	**Model 3**	**P-value**
	**OR (95%CI)**		**OR (95%CI)**		**OR (95%CI)**	
**PBDE28**	1.37 (1.05, 1.79)	**0.02**	1.39 (1.06, 1.83)	**0.02**	1.43 (1.10, 1.85)	**0.01**
**PBDE47**	1.35 (1.06, 1.71)	**0.02**	1.36 (1.07, 1.73)	**0.01**	1.39 (1.11, 1.75)	**0.005**
**PBDE85**	1.26 (1.04, 1.54)	**0.02**	1.29 (1.06, 1.57)	**0.01**	1.31 (1.09, 1.57)	**0.005**
**PBDE99**	1.23 (1.01, 1.50)	**0.04**	1.24 (1.01, 1.52)	**0.04**	1.27 (1.05, 1.54)	**0.02**
**PBDE100**	1.28 (1.02, 1.61)	**0.03**	1.30 (1.04, 1.62)	**0.02**	1.33 (1.08, 1.66)	**0.01**
**PBDE154**	1.24 (1.02, 1.50)	**0.03**	1.26 (1.04, 1.53)	**0.02**	1.29 (1.07, 1.55)	**0.01**
**PBDE183**	1.32 (1.07, 1.63)	**0.01**	1.37 (1.11, 1.69)	**0.003**	1.31 (1.04, 1.66)	**0.02**
**PBDE209**	1.10 (0.90, 1.35)	0.36	1.13 (0.93, 1.37)	0.22	1.12 (0.92, 1.36)	0.25
**PBB153**	1.36 (1.16, 1.60)	**< 0.001**	1.33 (1.12, 1.59)	**0.002**	1.25 (1.03, 1.53)	**0.03**

The bold values mean *p* < 0.05.

**Figure 2:**
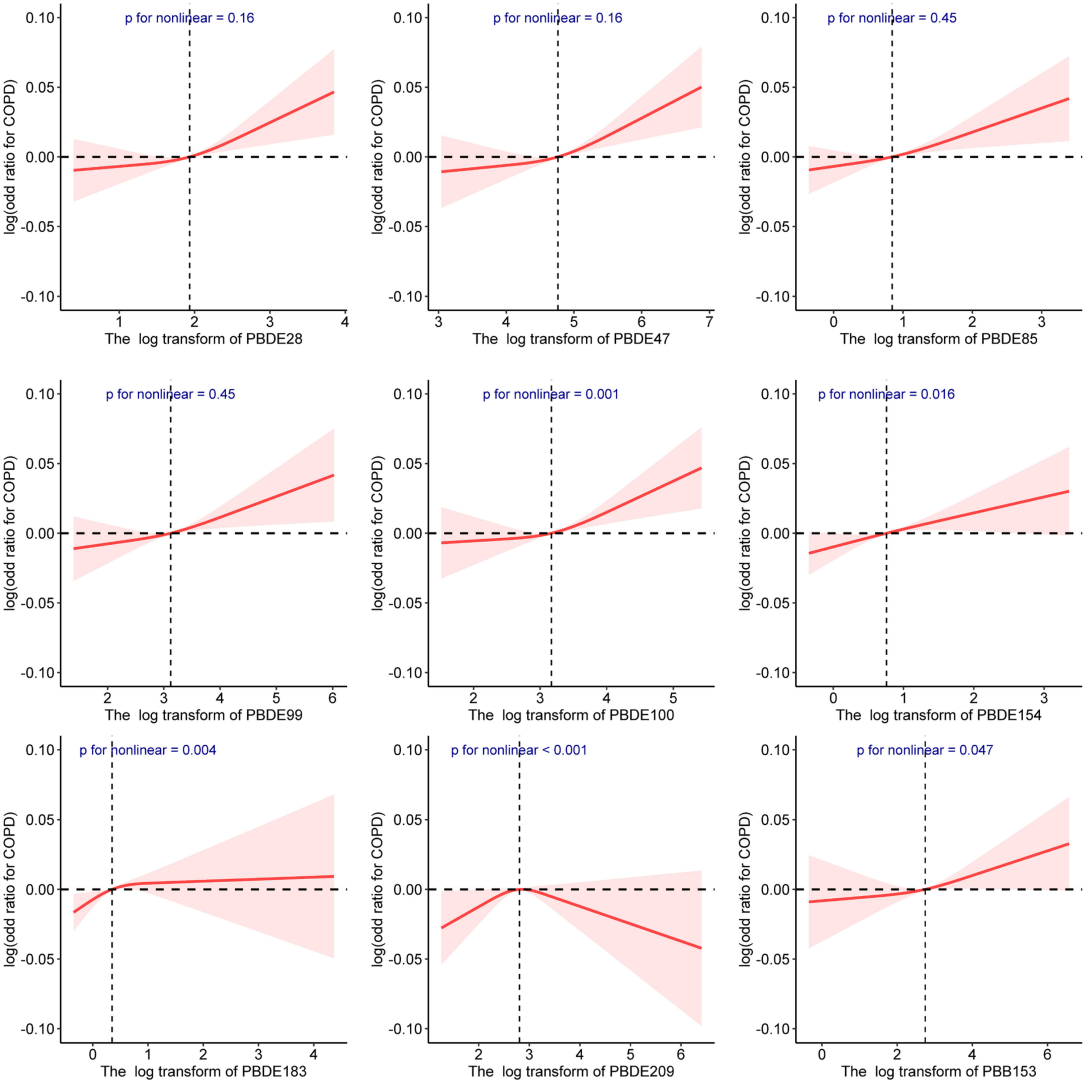
The non-linear associations between individual BFR and COPD by restricted cubic splines.

Restricted cubic splines curves displayed the potential nonlinearity between log-transformed concentrations of BFRs and the prevalence of COPD (Figure 2). PBDE-209 was significantly associated with CPOD in an inverted U-shape (*P* = 0.03). There were J-shaped associations between log-transformed PBDE-28, PBDE-47, PBDE-85, PBDE-99, PBDE-100, and PBB-153 concentrations with COPD prevalence, but those associations were insignificant. PBDE-154 had a relatively flat RCS fit. Stratified analysis by gender was displayed in Table 3. A significant interaction between being male and a high prevalence of COPD was observed for PBDE-28 (*P* for interaction <0.05), PBDE-47 (*P* for interaction <0.05), PBDE-85 (*P* for interaction <0.05), PBDE-99 (*P* for interaction <0.05), PBDE-100 (*P* for interaction <0.05), and PBB-153 (*P* for interaction <0.05). Besides, non-significant interaction existed between the young and middle-aged group with the elderly group (Table S2).

**Table 3:** Association between log-transformed serum BFRs and prevalence of COPD stratified by gender.

	**Subgroup**	**OR (95%CI)**	**P-value**	***P*** **_for interaction_**
**PBDE28**	Male	2.15 (1.58, 2.92)	<0.0001	<0.05
	Female	1.01 (0.72, 1.41)	0.95	
**PBDE47**	Male	2.08(1.62, 2.68)	<0.0001	<0.05
	Female	0.95 (0.69, 1.31)	0.77	
**PBDE85**	Male	1.81 (1.44, 2.27)	<0.0001	<0.05
	Female	0.93 (0.70, 1.24)	0.62	
**PBDE99**	Male	1.73 (1.41, 2.13)	<0.0001	<0.05
	Female	0.92 (0.70, 1.21)	0.56	
**PBDE100**	Male	1.98 (1.56, 2.53)	<0.0001	<0.05
	Female	0.88 (0.65, 1.20)	0.42	
**PBDE154**	Male	1.79 (1.44, 2.21)	<0.0001	<0.05
	Female	0.86 (0.64, 1.16)	0.31	
**PBDE183**	Male	1.59 (1.27, 2.01)	<0.001	0.09
	Female	1.04 (0.70, 1.53)	0.86	
**PBDE209**	Male	1.32 (1.05, 1.66)	0.02	0.10
	Female	0.94 (0.68, 1.31)	0.73	
**PBB153**	Male	1.15 (0.90, 1.48)	0.27	0.59
	Female	1.39 (1.03, 1.87)	0.03	

### Associations of mixture BFRs exposure with COPD prevalence

**Figure 3:**
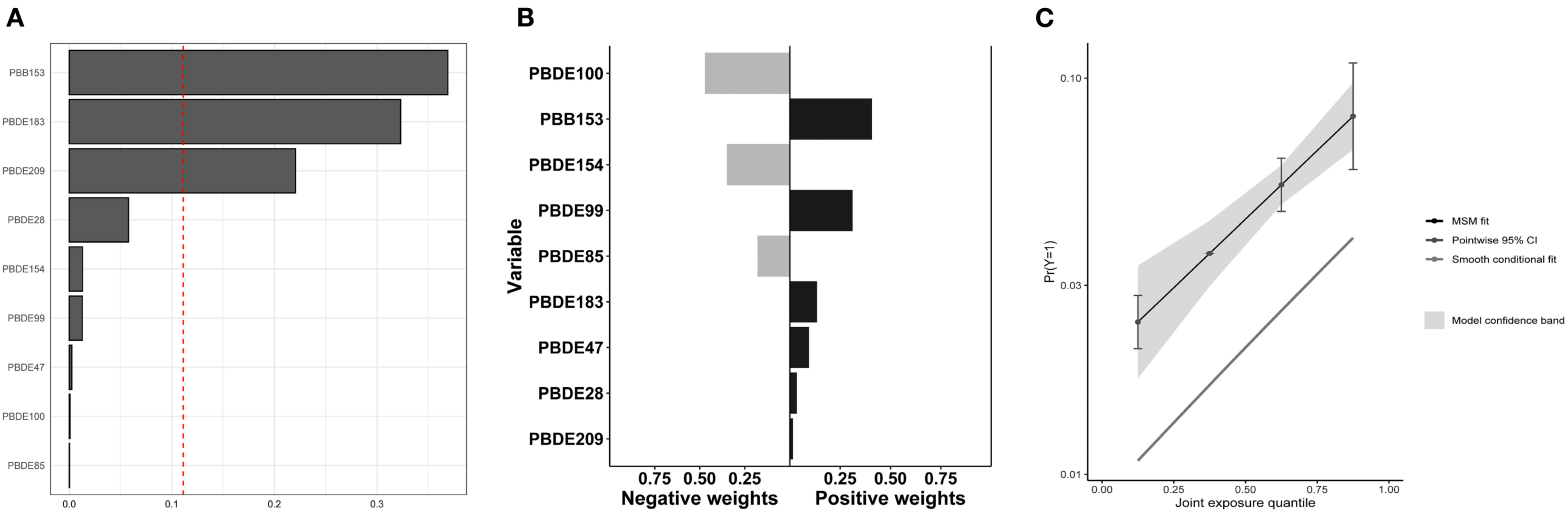
Associations of mixture BFRs exposure with COPD prevalence in all participants. **(A)** WQS regression index weights for COPD prevalence. **(B)** QGC regression index weights for COPD prevalence. **(C)** Joint effect (95% CI) for COPD prevalence in QGC analysis.

The associations between mixture BFRs exposure and COPD prevalence were analyzed using WQS regression and QGC analysis. With all confounding factors adjusted, the WQS index of mixture BFRs exposure was positively associated with COPD prevalence (OR: 1.40; 95% CI: 1.14–1.72, *P* = 0.002), and PBB153 (36.9%) made the most significant contribution (Figure 3A). Consistent with the WQS regression results, exposure to the mixture BFRs was positively associated with the prevalence of COPD in the QGC analysis (OR: 1.49; 95% CI: 1.27–1.74, *P* < 0.001), and PBB153 (40.8%) contributed the most (Figures 3B, C).

## Discussion

To the best of our knowledge, this is the first study to evaluate the associations between serum BFRs and COPD prevalence. This study indicated that PBDE-28, PBDE-47, PBDE-85, PBDE-99, PBDE-100, PBDE-154, PBDE-183, and PBB-153 were positively correlated with the prevalence of COPD in the survey-weighted generalized logistic regression model. Inverted u-shape associations were found between serum PBDE-209 and CPOD prevalence by RCS curves. The combined effect of the BFRs on CPOD prevalence was statistically significant in the WQS models and QGC analysis.

The WHO released a report in 2014 indicating that 3.7 million premature deaths globally were attributable to ambient air pollution (19). Between 2000 and 2018, a cohort study involving six regions of the United States found that long-term exposure to ambient air pollutants was significantly associated with increasing emphysema assessed quantitatively using CT imaging (20). Brominated flame retardants-contaminated air is a component of air pollution, which might be associated with the onset or development of COPD.

Despite the production ban, humans can still be exposed to BFRs in various pathways. The intake of PBDE-contaminated food, the ingestion of PBDE-contaminated dust, and the inhalation of PBDE-contaminated air appear to be the main pathways of PBDEs exposure (7, 21, 22). Vorkamp et al. identified that BDE47 and BDE99 were at high concentrations of 16.9 and 13.6 ng/g in domestic dust and 134 and 63.7 pg/m^3^ in residential indoor air (23). Outdoor air PBDE concentrations in America were reported to vary from 5 to more than 100 pg m^−3^ (24). Besides, dietary intake for PBDEs is estimated at 50 ng/d in the United States, mainly from dairy consumption, meat, and fish (25). Although the production of PBBs ceased in the United States in 1976, they can still be detected in environmental media or organisms for many years due to their environmental persistence and bioaccumulation. They can be enriched through breast milk transfer and the food chain (2, 26–28). In recent years, PBBs have been detected in biological media such as fish, birds, humans, and mussels, as well as in soil, water, and sediment (29–31).

Human health is impacted by BFRs that have thyrotoxicity, neurotoxicity, and reproductive developmental toxicity. Brominated flame retardants could disrupt thyroid homeostasis, including competing with thyroid hormones for binding to thyroid transport proteins, promoting thyroid hormone metabolism in the liver and brain, and altering thyroid hormone receptor activity (32). The mechanisms of the toxic effects of BFRs on the nervous system include affecting the release of neuroendocrine hormones, interfering with signal transduction pathways, affecting neurotransmitter transmission, altering the expression of essential proteins in the developing nervous system, and inducing apoptosis of neural cells (33, 34). Brominated flame retardants were also found to cause reproductive development toxicity by reducing sperm quality, interfering with hormone production, and damaging the structure of sexual organs (22, 35, 36).

Besides the above toxicity of BFRs, various researches have focused on the role of BFRs exposure on the respiratory system. In bronchial epithelial cell lines, exposure to PBDE-209 was found to reduce cell viability, increase LDH leakage, and promote inflammation (8). Besides, PBDE-47, PBDE-99, and PBDE-209 were shown to be involved in the dysregulation of oxidative stress, DNA damage, and associated gene expression (37). Albano et al. discovered that PBDE-47, PBDE-99, and PBDE-209 induced oxidative stress, inflammatory reaction, impairment of barrier integrity, and mucus production (38). Anzalone et al. discovered that PBDE-47, PBDE-99, and PBDE-209 increase inflammation by activating EZH2 methyltransferase (39). Zandona et al. confirmed that exposure to PBDEs (PBDE-28, PBDE-47, PBDE-99, PBDE-100, PBDE-153, PBDE-154, PBDE-183, and PBDE-209) caused membrane disruption, reactive oxygen species production, and cell apoptosis (4). Similar results were obtained in the animal experiments. The PBDEs (including PBDE-47, PBDE-85, and PBDE-99) could be effectively taken up and concentrated in the lung tissue of adult mice (9). In addition, mice exposed to PBDE-209 exhibited accumulation of PBDE-209 in lung tissue, widespread inflammation with perivascular, thickening and destruction of the alveolar wall, and enlargement of air spaces (38), which is a pathological manifestation of COPD.

There existed some limitations in our study. First, the self-reported COPD but not diagnosed by a physician might cause measurement bias, and a further study using physician-diagnosed COPD as the gold standard need to be explored. Second, due to the limitations of cross-sectional studies, it cannot be determined whether patients with COPD induced more BFRs exposure or whether BFRs exposure led to the development and progression of COPD. Third, despite enrolling as many confounding variables as possible, some potential confounding variables may still have influenced the results. Therefore, it is anticipated that large-scale and longitudinal research will clarify the causal relationship between BFRs exposure and COPD.

## Conclusion

Our study confirms that individual and mixture BFRs had positive associations with COPD, which needs to be further confirmed by longitudinal studies in larger-scale populations.

## Data availability statement

The datasets presented in this study can be found in online repositories. The names of the repository/repositories and accession number(s) can be found in the article/supplementary material.

## Author contributions

QW: conceptualization, methodology, and writing—reviewing and editing. LH: software and writing—original draft preparation. Both authors contributed to the article and approved the submitted version.

## Supplementary material

Figure S1 Flow chart for enrolled participants.

Table S1 Sensitivity analysis for the association between ln-transformed serum BFRs and risk of short sleep duration using the cotinine levels as the covariate.

Table S2 Association between log-transformed serum BFRs and prevalence of COPD stratified by age.

## References

[1] Collaborators GBDCRD. Prevalence and attributable health burden of chronic respiratory diseases, 1990–2017: a systematic analysis for the Global Burden of Disease Study 2017. Lancet Respir Med. (2020) 8:585–96. 10.1016/S2213-2600(20)30105-3 32526187 PMC7284317

[2] Alaee M Arias P Sjodin A Bergman A. An overview of commercially used brominated flame retardants, their applications, their use patterns in different countries/regions and possible modes of release. Environ Int. (2003) 29:683–9. 10.1016/S0160-4120(03)00121-1 12850087

[3] Rabe KF Watz H. Chronic obstructive pulmonary disease. Lancet. (2017) 389:1931–40. 10.1016/S0140-6736(17)31222-9 28513453

[4] Zandona A Jagic K Dvorscak M Madunic J Klincic D Katalinic M. PBDEs found in house dust impact human lung epithelial cell homeostasis. Toxics. (2022) 10:97. 10.3390/toxics10020097 35202283 PMC8874582

[5] de Wit CA. An overview of brominated flame retardants in the environment. Chemosphere. (2002) 46:583–624. 10.1016/S0045-6535(01)00225-9 11999784

[6] Watkins DJ McClean MD Fraser AJ Weinberg J Stapleton HM Sjodin A. Impact of dust from multiple microenvironments and diet on PentaBDE body burden. Environ Sci Technol. (2012) 46:1192–200. 10.1021/es203314e 22142368 PMC3268060

[7] Greeson KW Fowler KL Estave PM Kate Thompson S Wagner C Clayton Edenfield R. Detrimental effects of flame retardant, PBB_153_, exposure on sperm and future generations. Sci Rep. (2020) 10:8567. 10.1038/s41598-020-65593-x 32444626 PMC7244482

[8] Zhang Y Mao P Li G Hu J Yu Y An T. Delineation of 3D dose-time-toxicity in human pulmonary epithelial Beas-2B cells induced by decabromodiphenyl ether (BDE209). Environ Pollut. (2018) 243(Pt A):661–9. 10.1016/j.envpol.2018.09.047 30228062

[9] Darnerud PO Risberg S. Tissue localisation of tetra- and pentabromodiphenyl ether congeners (BDE-47,−85 and−99) in perinatal and adult C57BL mice. Chemosphere. (2006) 62:485–93. 10.1016/j.chemosphere.2005.04.004 15893803

[10] Feng Y Hu Q Meng G Wu X Zeng W Zhang X. Simulating long-term occupational exposure to decabrominated diphenyl ether using C57BL/6 mice: biodistribution and pathology. Chemosphere. (2015) 128:118–24. 10.1016/j.chemosphere.2015.01.012 25687576

[11] Tong Y Zhao X Li H Pei Y Ma P You J. Using homing pigeons to monitor atmospheric organic pollutants in a city heavily involving in coal mining industry. Chemosphere. (2022) 307(Pt 1):135679. 10.1016/j.chemosphere.2022.135679 35839993

[12] Koike E Yanagisawa R Takigami H Takano H. Penta- and octa-bromodiphenyl ethers promote proinflammatory protein expression in human bronchial epithelial cells *in vitro*. Toxicol In Vitro. (2014) 28:327–33. 10.1016/j.tiv.2013.10.014 24184330

[13] Wei W Ramalho O Mandin C. Modeling the bioaccessibility of inhaled semivolatile organic compounds in the human respiratory tract. Int J Hyg Environ Health. (2020) 224:113436. 10.1016/j.ijheh.2019.113436 31978732

[14] Albano GD Gagliardo RP Montalbano AM Profita M. Overview of the mechanisms of oxidative stress: impact in inflammation of the airway diseases. Antioxidants (Basel). (2022) 11:2237. 10.3390/antiox11112237 36421423 PMC9687037

[15] Kirkham PA Barnes PJ. Oxidative stress in COPD. Chest. (2013) 144:266–73. 10.1378/chest.12-2664 23880677

[16] Che Z Jia H Chen R Pan K Fan Z Su C. Associations between exposure to brominated flame retardants and metabolic syndrome and its components in U.S. adults. Sci Total Environ. (2023) 858(Pt 2):159935. 10.1016/j.scitotenv.2022.159935 36336051

[17] Carrico C Gennings C Wheeler DC Factor-Litvak P. Characterization of weighted quantile sum regression for highly correlated data in a risk analysis setting. J Agric Biol Environ Stat. (2015) 20:100–20. 10.1007/s13253-014-0180-3 30505142 PMC6261506

[18] Keil AP Buckley JP O'Brien KM Ferguson KK Zhao S White AJ. A quantile-based g-computation approach to addressing the effects of exposure mixtures. Environ Health Perspect. (2020) 128:047004. 10.1289/EHP5838 32255670 PMC7228100

[19] Whyand T Hurst JR Beckles M Caplin ME. Pollution and respiratory disease: can diet or supplements help? A review. Respir Res. (2018) 19:79. 10.1186/s12931-018-0785-0 29716592 PMC5930792

[20] Wang M Aaron CP Madrigano J Hoffman EA Angelini E Yang J. Association between long-term exposure to ambient air pollution and change in quantitatively assessed emphysema and lung function. JAMA. (2019) 322:546–56. 10.1001/jama.2019.10255 31408135 PMC6692674

[21] Hwang HM Park EK Young TM Hammock BD. Occurrence of endocrine-disrupting chemicals in indoor dust. Sci Total Environ. (2008) 404:26–35. 10.1016/j.scitotenv.2008.05.031 18632138 PMC2858057

[22] Wilford BH Thomas GO Jones KC Davison B Hurst DK. Decabromodiphenyl ether (deca-BDE) commercial mixture components, and other PBDEs, in airborne particles at a UK site. Environ Int. (2008) 34:412–9. 10.1016/j.envint.2007.09.007 17961649

[23] Vorkamp K Thomsen M Frederiksen M Pedersen M Knudsen LE. Polybrominated diphenyl ethers (PBDEs) in the indoor environment and associations with prenatal exposure. Environ Int. (2011) 37:1–10. 10.1016/j.envint.2010.06.001 20609475

[24] Besis A Samara C. Polybrominated diphenyl ethers (PBDEs) in the indoor and outdoor environments–a review on occurrence and human exposure. Environ Pollut. (2012) 169:217–29. 10.1016/j.envpol.2012.04.009 22578798

[25] Schecter A Haffner D Colacino J Patel K Papke O Opel M. Polybrominated diphenyl ethers (PBDEs) and hexabromocyclodecane (HBCD) in composite US food samples. Environ Health Perspect. (2010) 118:357–62. 10.1289/ehp.0901345 20064778 PMC2854763

[26] Bizkarguenaga E Ros O Iparraguirre A Navarro P Vallejo A Usobiaga A. Solid-phase extraction combined with large volume injection-programmable temperature vaporization-gas chromatography-mass spectrometry for the multiresidue determination of priority and emerging organic pollutants in wastewater. J Chromatogr A. (2012) 1247:104–17. 10.1016/j.chroma.2012.05.022 22673809

[27] Sjodin A Wong LY Jones RS Park A Zhang Y Hodge C. Serum concentrations of polybrominated diphenyl ethers (PBDEs) and polybrominated biphenyl (PBB) in the United States population: 2003–2004. Environ Sci Technol. (2008) 42:1377–84. 10.1021/es702451p 18351120

[28] Zhihua L Panton S Marshall L Fernandes A Rose M Smith F. Spatial analysis of polybrominated diphenylethers (PBDEs) and polybrominated biphenyls (PBBs) in fish collected from UK and proximate marine waters. Chemosphere. (2018) 195:727–34. 10.1016/j.chemosphere.2017.11.114 29289018

[29] Chang CJ Terrell ML Marcus M Marder ME Panuwet P Ryan PB. Serum concentrations of polybrominated biphenyls (PBBs), polychlorinated biphenyls (PCBs) and polybrominated diphenyl ethers (PBDEs) in the Michigan PBB Registry 40 years after the PBB contamination incident. Environ Int. (2020) 137:105526. 10.1016/j.envint.2020.105526 32062441 PMC7201813

[30] Mwangi JK Lee WJ Wang LC Sung PJ Fang LS Lee YY. Persistent organic pollutants in the Antarctic coastal environment and their bioaccumulation in penguins. Environ Pollut. (2016) 216:924–34. 10.1016/j.envpol.2016.07.001 27400905

[31] Falandysz J Smith F Fernandes AR. Dioxin-like polybrominated biphenyls (PBBs) and ortho-substituted PBBs in edible cod (*Gadus morhua*) liver oils and canned cod livers. Chemosphere. (2020) 248:126109. 10.1016/j.chemosphere.2020.126109 32041076

[32] Wu Z He C Han W Song J Li H Zhang Y. Exposure pathways, levels and toxicity of polybrominated diphenyl ethers in humans: a review. Environ Res. (2020) 187:109531. 10.1016/j.envres.2020.109531 32454306

[33] Costa LG de Laat R Tagliaferri S Pellacani C. A mechanistic view of polybrominated diphenyl ether (PBDE) developmental neurotoxicity. Toxicol Lett. (2014) 230:282–94. 10.1016/j.toxlet.2013.11.011 24270005 PMC4028440

[34] Eriksson P Jakobsson E Fredriksson A. Brominated flame retardants: a novel class of developmental neurotoxicants in our environment? Environ Health Perspect. (2001) 109:903–8. 10.1289/ehp.01109903 11673118 PMC1240439

[35] Sarkar D Singh SK. Inhibition of testicular steroidogenesis and impaired differentiation of Sertoli cells in peripubertal mice offspring following maternal exposure to BDE-209 during lactation suppress germ cell proliferation. Toxicol Lett. (2018) 290:83–96. 10.1016/j.toxlet.2018.03.026 29578053

[36] Zhai J Geng X Ding T Li J Tang J Chen D. An increase of estrogen receptor alpha protein level regulates BDE-209-mediated blood-testis barrier disruption during spermatogenesis in F1 mice. Environ Sci Pollut Res Int. (2019) 26:4801–20. 10.1007/s11356-018-3784-2 30565106

[37] Montalbano AM Albano GD Anzalone G Moscato M Gagliardo R Di Sano C. Cytotoxic and genotoxic effects of the flame retardants (PBDE-47, PBDE-99 and PBDE-209) in human bronchial epithelial cells. Chemosphere. (2020) 245:125600. 10.1016/j.chemosphere.2019.125600 31864052

[38] Albano GD Moscato M Montalbano AM Anzalone G Gagliardo R Bonanno A. Can PBDEs affect the pathophysiologic complex of epithelium in lung diseases? Chemosphere. (2020) 241:125087. 10.1016/j.chemosphere.2019.125087 31622892

[39] Anzalone G Moscato M Montalbano AM Albano GD Gagliardo R Marchese R. PBDEs affect inflammatory and oncosuppressive mechanisms via the EZH2 methyltransferase in airway epithelial cells. Life Sci. (2021) 282:119827. 10.1016/j.lfs.2021.119827 34273373

