# Supplementary material for: Associations of brominated flame retardants exposure with chronic obstructive pulmonary disease: A US population-based cross-sectional analysis
Source: Front Public Health. 2023 Mar 10;11:1138811. doi: 10.3389/fpubh.2023.1138811 (PMC10036799; doi:10.3389/fpubh.2023.1138811)
Supplement: Figure S1 — Flow chart for enrolled participants. [file Data_Sheet_1.pdf]

Table S1. Sensitivity analysis for the association between ln-transformed serum BFRs and risk of short sleep duration using the cotinine levels as the covariate.

Chemicals	Ln-transformed serum BFRs OR (95%CI)	Quantile 1 OR (95%CI)	Quantile 2 OR (95%CI)	Quantile 3 OR (95%CI)	Quantile 4 OR (95%CI)	$p_{\text{for trend}}$
**PBDE28**	1.35(1.05, 1.75)	Reference	1.32(0.73, 2.38)	1.61(0.91, 2.84)	2.00(1.18, 3.38)	**0.003**
**PBDE47**	1.33(1.05, 1.68)	Reference	1.12(0.65, 1.91)	1.67(1.02, 2.74)	1.83(1.15, 2.93)	**0.002**
**PBDE85**	1.26(1.04, 1.52)	Reference	1.24(0.85, 1.80)	1.45(0.94, 2.22)	1.85(1.21, 2.84)	**0.005**
**PBDE99**	1.22(1.00, 1.49)	Reference	1.01(0.62, 1.67)	1.42(0.89, 2.27)	1.81(1.16, 2.81)	**0.004**
**PBDE100**	1.28(1.03, 1.58)	Reference	0.89(0.58, 1.35)	1.47(0.91, 2.37)	1.61(1.05, 2.48)	**0.006**
**PBDE154**	1.24(1.03, 1.50)	Reference	1.18(0.76, 1.83)	1.56(0.96, 2.54)	1.68(1.08, 2.60)	**0.008**
**PBDE183**	1.30(1.02, 1.64)	Reference	0.72(0.49, 1.06)	1.63(1.09, 2.44)	1.40(0.87, 2.26)	**0.01**
**PBDE209**	1.09(0.89, 1.34)	Reference	1.20(0.73, 1.97)	1.82(1.31, 2.54)	1.50(1.00, 2.26)	**0.008**
**PBB153**	1.26(1.04, 1.52)	Reference	4.60(1.88,11.29)	5.35(2.05,13.95)	6.37(2.45,16.59)	**0.02**

Notes: Model was adjusted for age, gender, race/ethnicity, FIR, BMI, cotinine levels, physical activity, hypertension and diabetes.

Table S2. Association between ln-transformed serum BFRs and prevalence of COPD stratified by age.

	Subgroup	OR (95%CI)	P value	P for interaction
**PBDE28**	Age > 60	1.78(1.35, 2.35)	<0.001	0.57
	Age ≤ 60	1.54(1.06, 2.23)	0.02	
**PBDE47**	Age > 60	1.69(1.35,2.11)	<0.0001	0.26
	Age ≤ 60	1.36(0.95, 1.94)	0.09	
**PBDE85**	Age > 60	1.56(1.30,1.88)	<0.0001	0.15
	Age ≤ 60	1.23(0.92, 1.64)	0.16	
**PBDE99**	Age > 60	1.42(1.19,1.70)	<0.001	0.46
	Age ≤ 60	1.28(0.94, 1.73)	0.11	
**PBDE100**	Age > 60	1.63(1.31,2.02)	<0.0001	0.13
	Age ≤ 60	1.22(0.87, 1.71)	0.25	
**PBDE154**	Age > 60	1.44(1.22,1.70)	<0.0001	0.48
	Age ≤ 60	1.31(0.97, 1.76)	0.07	
**PBDE183**	Age > 60	1.26(0.85,1.87)	0.25	0.87
	Age ≤ 60	1.42(1.05, 1.91)	0.02	
**PBDE209**	Age > 60	1.08(0.82,1.43)	0.59	0.76
	Age ≤ 60	1.27(0.92, 1.75)	0.14	
**PBB153**	Age > 60	1.19(0.89,1.59)	0.25	0.03
	Age ≤ 60	1.70(1.41, 2.05)	<0.0001	

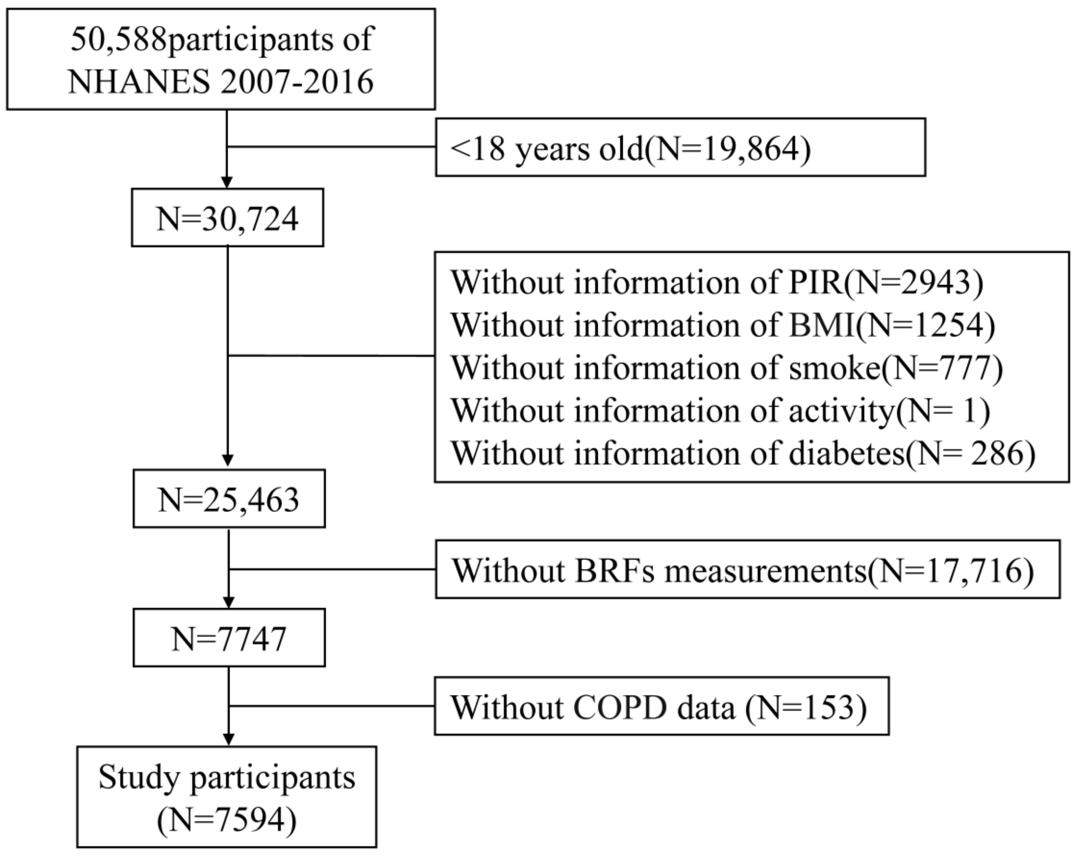
50,588participants of NHANES 2007-2016
<18 years old(N=19,864)
N=30,724
Without information of PIR(N=2943)
Without information of BMI(N=1254)
Without information of smoke(N=777)
Without information of activity(N= 1)
Without information of diabetes(N= 286)
N=25,463
Without BRFs measurements(N=17,716)
N=7747
Without COPD data (N=153)
Study participants (N=7594)
